# Discrimination of serum metabolomics profiles in infants with sepsis, based on liquid chromatography-mass spectrometer

**DOI:** 10.1186/s12879-023-07983-w

**Published:** 2023-01-23

**Authors:** Li Wang, Xinyi Cha, Zhongxiao Zhang, Jihong Qian

**Affiliations:** 1 Clinic and Research Center of Tuberculosis, Shanghai Pulmonary Hospital, Institute for Advanced Study, Tongji University School of Medicine, Shanghai, China; 2 Department of Neonatology, Xinhua Hospital, School of Medicine, Shanghai Jiaotong University, Shanghai, China; 3 Hongqiao International Institute of Medicine, Tongren Hospital, School of Medicine, Shanghai Jiaotong University, Shanghai, China

**Keywords:** Sepsis, Infant, Metabolomics, Biomarker

## Abstract

Sepsis is one of the most important problems to be addressed in pediatrics, characterized by insidious onset, rapid progression, and high rates of severe infection and even mortality. Biomarkers with high sensitivity and robustness are urgently required for the early diagnosis of infant sepsis. Serum metabolomic approaches based on liquid chromatography-mass spectrometry were used to analyze the samples from 30 infants with sepsis at an early stage and 30 infants with noninfectious diseases. Multivariate statistical analysis was used to screen for differential metabolites and ROC curves were generated to find potential biomarkers. Six metabolites, including phosphatidic acid (PA (8:0/14:0)), phosphatidyl ethanolamine (PE (16:0/18:2(9Z,12Z))), cytidine 5'-diphosphocholine (CDP-CHO), sphingomyelin (SM (d18:0/16:1(9Z))), prolylhydroxyproline and phosphorylcholine (P-CHO), were identified between the two groups. ROC curve analysis showed that prolylhydroxyproline (AUC = 0.832) had potential diagnostic values for infant sepsis. The AUC value was 0.859 (CI: 0.764, 0.954) in the combined model. Prolylhydroxyproline were found to be correlated with CRP and PCT levels, while PE and CDP-CHO associated with PCT levels. Pathway analysis indicated that glycerophospholipid metabolism, aminoacyl-tRNA biosynthesis and necroptosis pathways played important roles in infant sepsis. Network analysis showed that the differential metabolites were linked to ERK/ MAPK, NF-κB, AMPK, mTOR, and other classical inflammatory and metabolic signaling pathways. This study identified serum metabolite profiles and three metabolites as potential biomarkers in infants with sepsis. The findings will help improve the early diagnosis of sepsis in infants.

**Supplementary Information:**

The online version contains supplementary material available at 10.1186/s12879-023-07983-w.

## Background

Sepsis is an inflammatory response triggered by toxins from various pathogenic microorganisms in the circulatory system that threatens the life of infants, especially neonates [1]. On a global scale, sepsis contributes to morbidity and mortality and was identified by the WHO as a top healthcare priority for the next decade. Neonatal sepsis is estimated to affect 2202 (95% CI: 1099–4360) per 100,000 live births, with a mortality rate of 11% to 19% [2].

Due to the frequent occurrence of non-infectious diseases resembling sepsis, the diagnosis of infantile or neonatal sepsis is very complicated, and conventional examination methods are not entirely reliable [3]. Although blood culture is the gold standard for the diagnosis of sepsis, the limited volume of blood samples in neonates impairs its accuracy and sensitivity [4]. In addition, the time required for blood culture is not conducive to the early diagnosis of sepsis, leading to delays in treatment. Moreover, other commonly used laboratory markers in clinical practice, such as peripheral blood leukocytes, procalcitonin (PCT), and C-reactive protein (CRP), have limitations in terms of sensitivity and specificity. Therefore, clinicians urgently need accurate biomarkers to improve the diagnosis of neonatal sepsis.

Metabolomics, which quantitatively detects biochemical responses to pathophysiological stimuli or genetic modifications, has emerged as a new methodology in biological research in recent years. It has been shown in studies that metabolomics may be able to serve as biomarkers in pneumonia and hepatitis B virus infection in children [5, 6]. Additionally, Pandovan MG used metabolomics methods to identify candidates related to the diagnosis and prognosis of sepsis in adults[7]. Clinical studies have found an association between lipid metabolism and sepsis, a variety of lipid profiles are altered in adult patients with sepsis, including high-density lipoproteins [8], cholesterol [9], triglycerides [10, 11] and acylcarnitine [12]. Furthermore, five lipidomic-related metabolites, sphingolipids, acylcarnitines, fatty acid esters, and two glycerol phosphocholines, have been validated as diagnostic biomarkers for sepsis in adults [13]. Metabolites are potential biomarkers for sepsis. Although changes in metabolites profiles in adult patients have raised the interest of scientists regarding the role of metabolism in sepsis, little is known about these changes in infants and neonates.

In this study, liquid chromatography-mass spectrometry (LC-MS) was used to identify differential metabolites in serum samples from infants with sepsis. These results provide valuable evidence for the accurate identification of sepsis at an early stage in infants.

## Methods

### Patient recruitment

This study included 60 patients admitted to our hospital between May 2019 and May 2020. 30 infants with signs of infection and confirmed sepsis by a subsequent series of tests [1] (including routine blood test, CRP, PCT, arterial blood gas analysis, blood culture and chest x-ray, etc.) were selected as the sepsis group, and 30 infants with non-infectious diseases were selected as the control group. All infants were born after a full-term pregnancy. Both groups were matched according to gender, weight at admission, age in days, with all subjects being less than two months old. The exclusion criteria were as follows: (1) infants who used antibiotics for more than two weeks before admission; (2) infants who received total parenteral nutrition support for more than seven days before admission; (3) infants with a diagnosis of infantile cholestasis or congenital heart disease; (4) infants with confirmed or suspected congenital metabolic disease; (5) Mothers with a history of gestational diabetes, intrahepatic cholestasis, or abnormal liver function. All methods were carried out in accordance with relevant guidelines and regulations.

### Sample collection and preparation

Whole blood samples were collected into serum separator tube within 24 h following admission. The samples were centrifuged at 3000 rpm for 15 min at 4 ℃. The supernatant was then removed into a 1.5 mL centrifuge tube for LC-MS detection. First, the solution was vortexed for 2 min after mixing 100 µL of serum with 300 µL methanol solution (containing 5 µg/ml L-2-chloro-phenylalanine as an internal standard). After centrifugation at 13,000 rpm at 4 °C for 10 min, 200 µl supernatant was collected. Finally, an equal volume of serum from each sample was mixed to prepare a quality control (QC) sample.

### LC-MS

The LC-MS analysis was carried out using an Ultimate 3000 ultra-high-performance liquid chromatograph (Thermo Fisher, USA) coupled with an Orbitrap Elite mass spectrometer (Thermo Fisher, USA). Chromatographic column: Kinetex C18 (100 × 2.1 mm, 1.9 μm). Mobile phase: A-0.1% formic acid solution, B-acetonitrile (0.1% formic acid); flow rate: 0.4 ml/min; column temperature, 25 °C; post time, 5 min; injection volume, 3 µL. Optimized chromatographic gradient: 0–2 min, 5% B; 2–− 13 min, 5–95% B; 13–15 min, 95% B. The post time was set to 5 min to equilibrate the system. The mass spectrometer operates in both positive-ion and negative-ion modes. The optimized parameters are as follows: positive mode, Heater Temp 300 °C, Sheath Gas Flow rate 45 arb, Aux Gas Flow Rate 15 arb, Sweep Gas Flow Rate 1 arb, spray voltage 3.0 kV, Capillary Temp 350 °C, S-Lens RF Level 30%, Scan ranges: 200–1500. Negative mode, Heater Temp 300 °C, Sheath Gas Flow rate 45 arb, Aux Gas Flow Rate 15 arb, Sweep Gas Flow Rate 1 arb, spray voltage 2.5 kV, Capillary Temp 350 °C, S-Lens RF Level 60%, Scan ranges: 50–2100 Da. After detection, the accurate ion mass was inputted into the human metabolome database (HMDB), lipid search, Metlin, MoNA and massbank databases through matching the exact molecular weight. MS1/MS2 fragment ions were automatically searched. For confirming the structure of the compound, internal standard metabolite library was used to match the exact mass, fragment ion mass, and retention time.

### Statistical analysis

Data were collected using the Thermo Xcalibur 2.2 software (Thermo Scientific, San Jose, USA) and edited with Microsoft Excel (2007). Continuous variables were expressed by mean ± standard deviation (‾x ± sd), and non-normal distribution was represented by M (P25, P75). Comparison of means between groups was performed using independent samples T-test for normally distributed data and Mann–Whitney U-test for non-uniformly distributed data. Categorical variables are presented as N (%) and compared using the chi-square test or Fisher exact test. The Simca-P software (version 11.0; Umetrics, Sweden) was used to perform partial least squares discriminant analysis (PLS-DA). The false discovery rate (FDR) was used for multiple testing adjustment. Pathway enrichment analysis of differential metabolites was performed using a hypergeometric test and topological analysis with the aid of the online database KEGG (Kyoto Encyclopedia of Genes and Genomes, www.kegg.jp/kegg/kegg1.html) pathway analysis. Bioinformatics analysis of differential metabolites was conducted using ingenuity pathway analysis (IPA) software. Clinical data were analyzed using SPSS Statistics 25 (version 25.0.0.1, IBM, USA). Statistical significance was set at P < 0.05.

## Results

### Characteristics of the subjects

60 patients (30 in the sepsis group and 30 in the control group) were enrolled in the study. Most of neonates in control group were admitted for neonatal jaundice, atrial septal defect, and umbilical hernias, who needed to evaluate in details or accepted treatment. The two groups were comparable in terms of baseline characteristics (P > 0.05), including sex, mode of birth, day age of blood draw, gestational age, birth weight, body weight at the time of collection, the proportion of exclusive breastfeeding, premature rupture of membranes (PROM) ≥ 18 h, mothers with fever at birth, gestational diabetes, gestational hypertension (Table 1). In the sepsis group, eleven positive blood culture cases were identified, three *Staphylococcus epidermidis*, two *Escherichia coli*, two *Enterococcus faecium*, two *Klebsiella pneumoniae*, one *Staphylococcus aureus,* and one *Streptococcus agalactiae*. In addition, the sepsis group showed significantly higher levels of white blood cell count (WBC), percentage of neutrophils (N %), PCT, and CRP levels than the control group (P < 0.05). All the cases in sepsis group survived, and only one baby from sepsis group who transferred to NICU and treated with endotracheal intubation and mechanical ventilation was discharged after 20 days hospital stay.

**Table 1:** The demographic and perinatal characteristics, and laboratory findings of the subjects

Descriptive variable	Sepsis (n = 30)	Control (n = 30)	*P*
Male sex (%)	16 (53.33)	18 (60.00)	0.80
Cesarean (%)	14 (46.67)	12 (40.00)	0.60
Age of blood collection [days]	20.00 [6.00]	23.50 [8.25]	0.95
Gestational age [weeks]	39.00 [36.57]	39.00 [38.29]	0.95
Birth weight (kg)	3.41 (0.67)	3.33 (0.41)	0.61
Weight^1^ (kg)	4.22 (1.10)	3.98 (0.78)	0.33
Breastfed (%)	7 (23.33)	11 (36.67)	0.40
PROM ≥ 18 h (%)	1 (3.33)	0 (0)	1.00
Mother with GDM (%)	1 (3.33)	0 (0)	1.00
WBC [10^9^/L]	12.62 [9.03]	10.27 [8.25]	0.04
Percentage of neutrophils (%)	57.58 (19.89)	40.11 (15.76)	< 0.001
CRP [mg/L]	23.5 [0.71]	0.71 [0.71]	< 0.001
PCT [ng/mL]	1.39 [0.34]	0.10 [0.08]	< 0.001

Normally distributed numerical data are reported as mean (standard deviation), whereas non-normally distributed data are reported as median [interquartile range] and categorical data are reported as number of cases (percentage) relative to the reference group

^1^Weight of the patients when admission

### Metabolomic profiling of serum samples

**Fig. 1:**
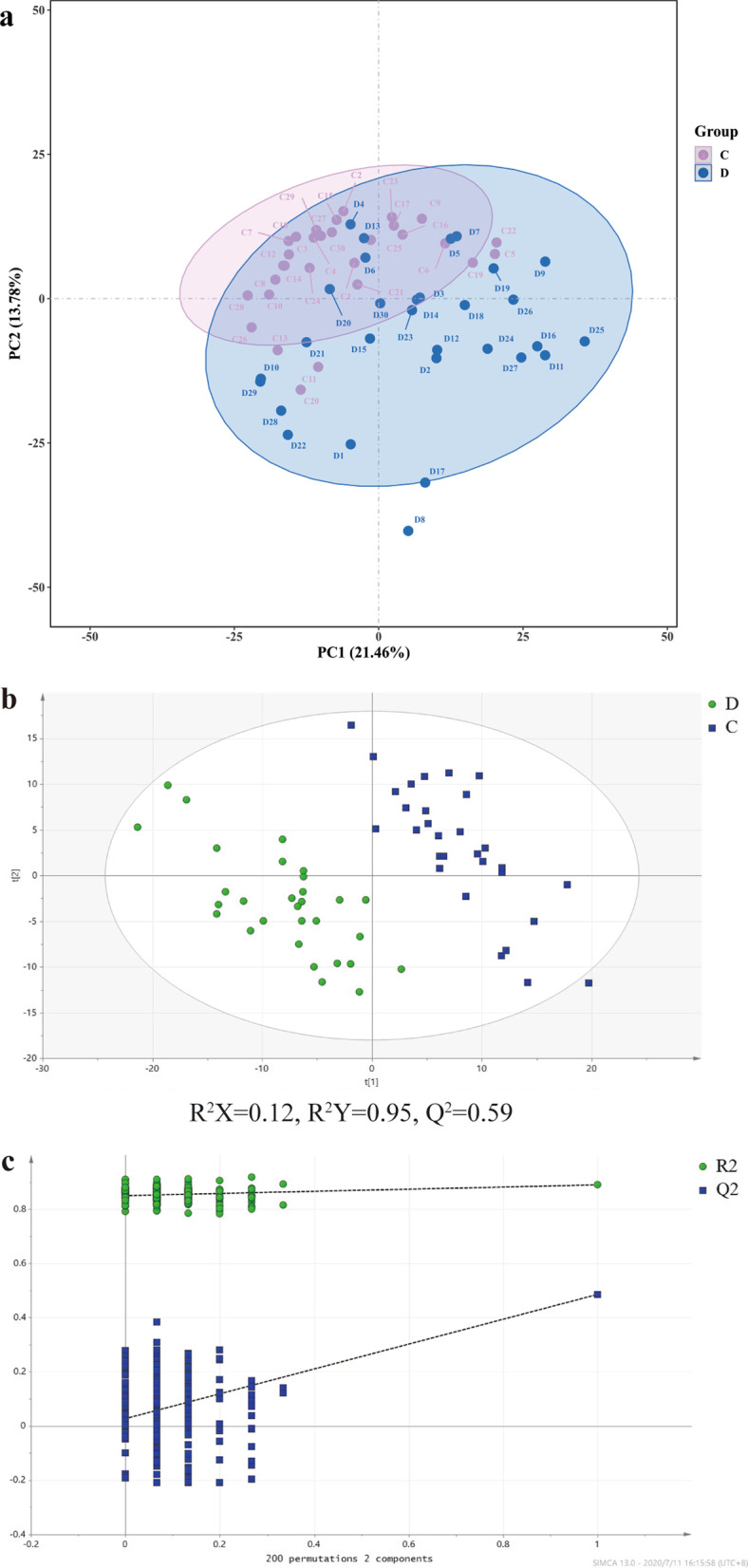
The score plot of the PLS-DA model in the positive mode. **a** The PCA score map, **b** The PLS-DA score map and **c** permutation test in the positive mode. The letter D and symbol ● represent the sepsis group; the letter C and symbol ■ represent the control group

**Fig. 2:**
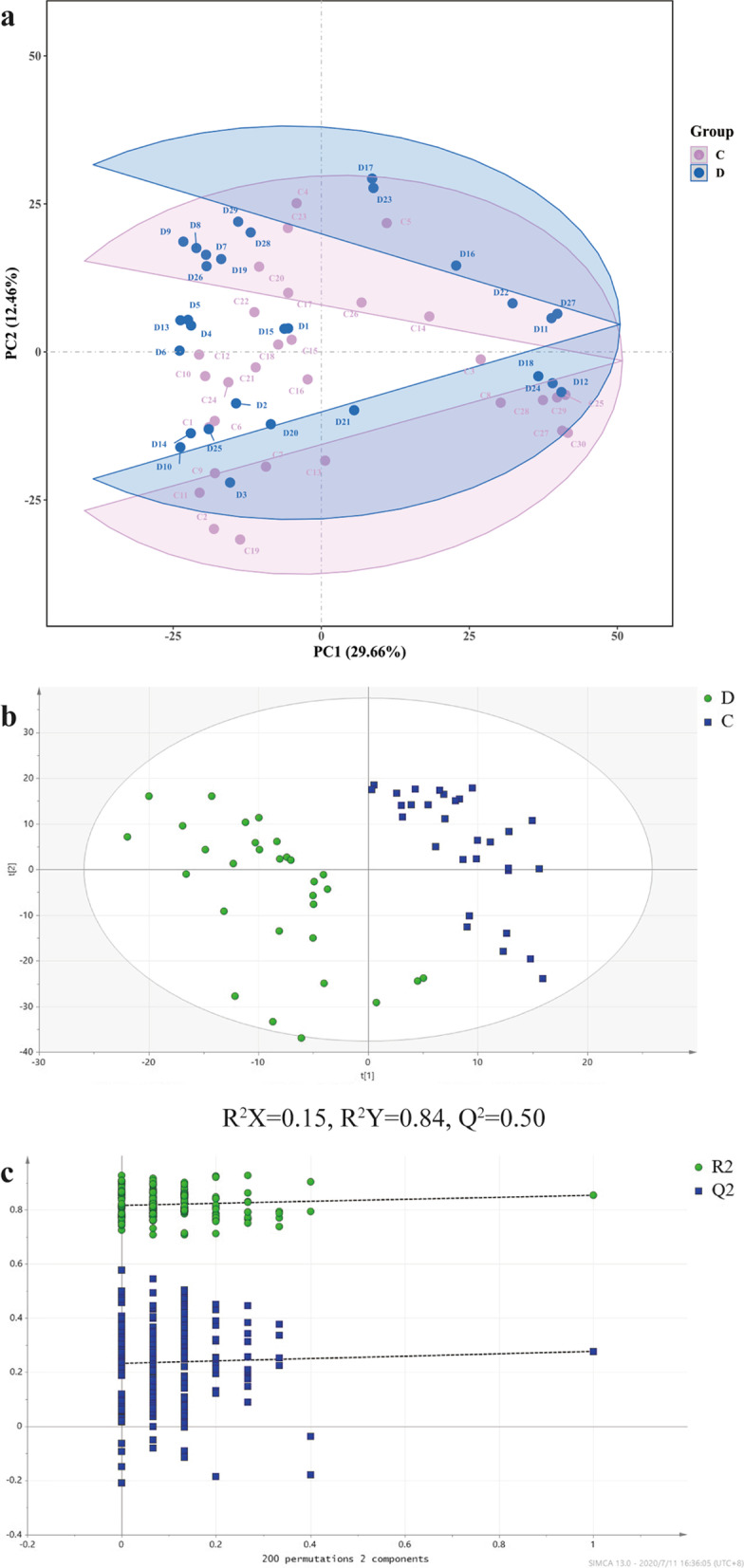
The score plot of the PLS-DA model in the negative mode. **a** The PCA score map, **b** The PLS-DA score map and **c** permutation test in the negative mode. The letter D and symbol ● represent the sepsis group; the letter C and symbol ■ represent the control group

Volcano plots and heatmaps demonstrated differences in these metabolites between sepsis and control groups (Additional file 4: Fig. S1). The PLS-DA score plot was calculated via 200 times cross-validation based on the positive/negative ion mode. The PLS-DA score plot indicated an obvious separation trend between groups. Furthermore, the R^2^X, R^2^Y and Q^2^ values implied that this model had good stability and predictability (Figs. 1, 2).

**Fig. 3:**
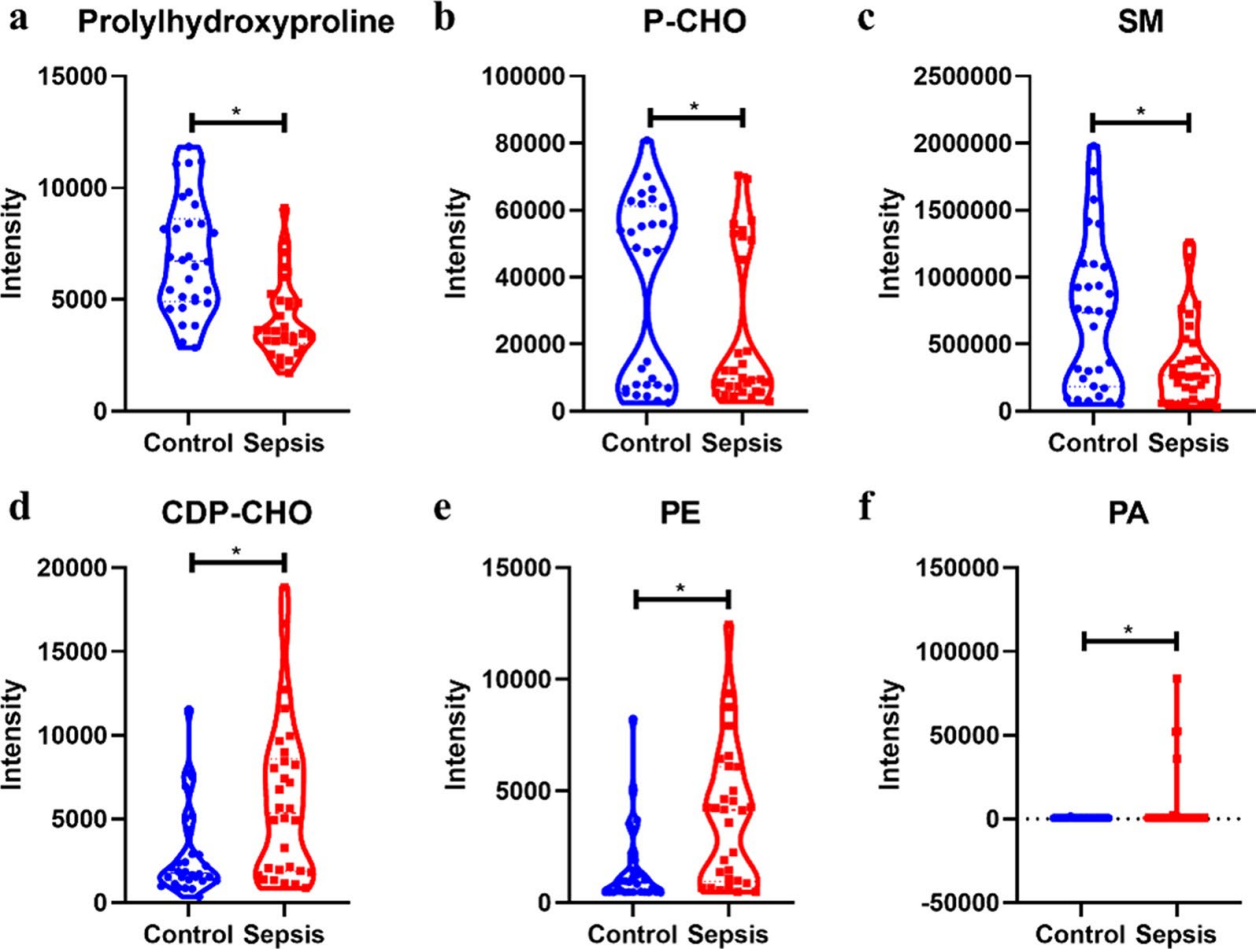
Violin plot of differential metabolites. **a** Prolylhydroxyproline; **b** P-CHO; (**c**)SM (d18:0/16:1(9Z)); **d** CDP-CHO; **e** PE (16:0/18:2(9Z,12Z)); **f** PA(8:0/14:0)

26 differential metabolites were identified (VIP > 1 and P < 0.05, Additional file 1: Table S1), 6 of which significantly differential metabolites between the two groups through PLS-DA model (VIP > 1, *FDR* < 0.05, and Log_2_FC > 0.58 or Log_2_FC < − 0.58, Table 2). Compared with the control group, three metabolites, phosphatidic acid (PA (8:0/14:0)), phosphatidyl ethanolamine (PE (16:0/18:2(9Z,12Z))), and cytidine 5'-diphosphocholine (CDP-CHO), were upregulated in the sepsis group, while the levels of other three metabolites, including sphingomyelin (SM (d18:0/16:1(9Z))), prolylhydroxyproline, and phosphorylcholine(P-CHO), were decreased in the sepsis group (Fig. 3a–f). The means, Standard Deviations, medians, and interquartile ranges for the markers are provided in the Additional file 2: Table S2. The MS2 fragment ion spectra of the differential metabolites are presented in Additional file 5: Appendix S1, with four metabolites confirmed by standard compounds.

**Table 2:** Differential metabolites (sepsis group versus control group)

Name	RT [min]	m/z	VIP	Log_2_FC	*FDR*
Prolylhydroxyproline	1.44	228.11	2.52	− 0.77	0.0002
SM(d18:0/16:1(9Z))	9.67	702.57	1.99	− 0.99	0.0103
PE(16:0/18:2(9Z,12Z))	9.2	715.51	1.97	1.37	0.0023
Cytidine 5'-diphosphocholine	7.54	541.38	1.8	1.09	0.0066
Phosphocholine	8.66	183.07	1.26	− 0.67	0.0455
PA(8:0/14:0)	16.69	508.32	1.16	3.84	0.0495

### ROC analysis of metabolites

**Fig. 4:**
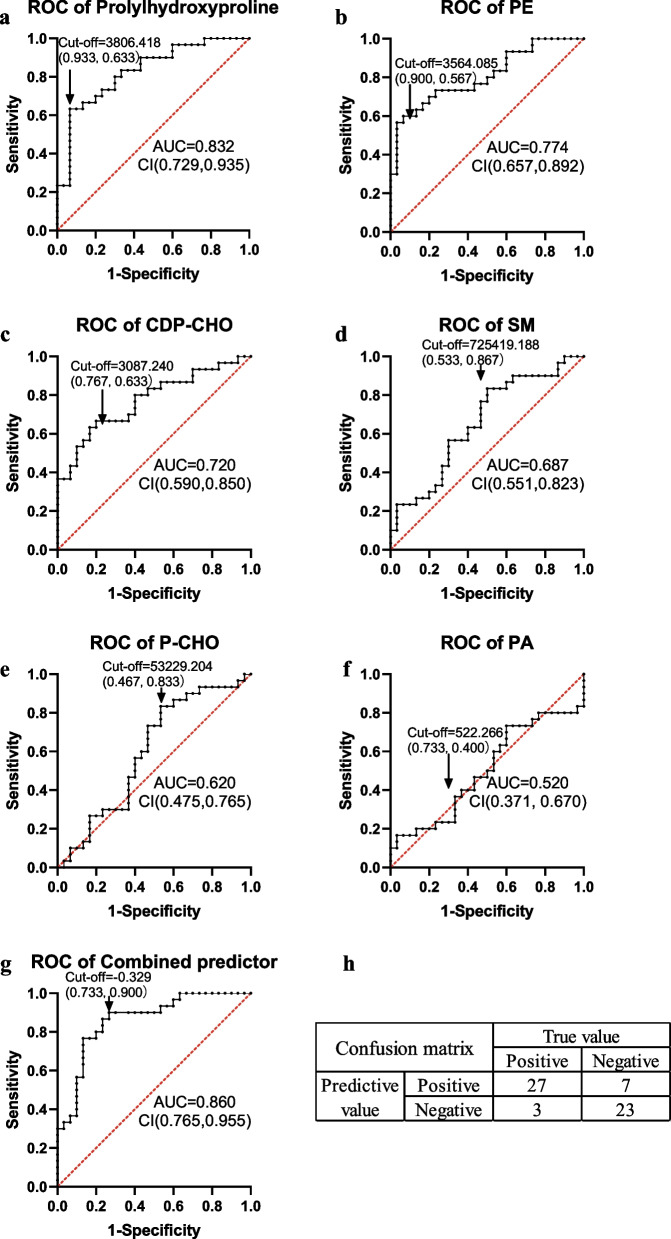
ROC curves of differential metabolites. **a** Prolylhydroxyproline; **b** PE(16:0/18:2(9Z,12Z)); **c** CDP-CHO; **d** SM(d18:0/16:1(9Z)); **e** P-CHO; **f** PA (8:0/14:0); **g** combined predictor of prolylhydroxyproline, PE (16:0/18:2(9Z,12Z)) and CDP-CHO. **h** confusion matrix of the combined predictor

For these 6 lipid metabolites, ROC curves were performed, with cut-off values calculated and confusion matrices obtained (Fig. 4 a-f, Additional file 3: Table S3). ROC curve analysis illustrated that the diagnostic ability of three metabolites for sepsis was greater than 0.7. The AUC of prolylhydroxyproline was greater than 0.8. Subsequently, a combined predictor of the three metabolites was evaluated by ROC analysis, which had AUC of 0.860 (CI: 0.765, 0.955). This combined predictor has a high sensitivity (0.900, Fig. 4 g, h) and is suitable for early screening of infant sepsis.

### Correlation analysis of metabolites and clinic indicators

PE(16:0/18:2(9Z,12Z)) and CDP-CHO were both involved in glycerophospholipid metabolism pathway and phospholipid biosynthesis, while prolylhydroxyproline was a relatively independent compound. Therefore, spearman correlation analysis explored the correlation between prolylhydroxyproline and CRP, PCT and partial correlations used for PE (16:0/18:2(9Z,12Z)), CDP-CHO.

Interestingly, the results indicated that levels of prolylhydroxyproline were negatively correlated with levels of CRP (r = − 0.27, P = 0.03) and PCT (r = − 0.31, P = 0.02). Significant negative correlations were observed between the levels of PE (16:0/18:2(9Z,12Z)) and PCT (r = − 0.34, P = 0.008). Additionally, there was a significant positive correlation between serum CDP-CHO levels and the PCT levels (r = 0.48, P < 0.001) (Table 3).

**Table 3:** Correlation analysis of 3 metabolites with CRP and PCT

	CRP		PCT	
	r	*P*	r	*P*
PE (16:0/18:2(9Z,12Z))*	− 0.005	0.97	− 0.34	0.008
CDP-CHO*	0.07	0.59	0.48	< 0.001
Prolylhydroxyproline	− 0.27	0.03	− 0.31	0.02

*r and *p* from partial correlation analysis

### Pathway analysis

Pathway enrichment analysis was used to further investigate observed variations and identify crucial molecules among these significantly differential metabolites. As shown in Table 4, the differential metabolites identified in this study were related to glycerophospholipid metabolism, aminoacyl-tRNA biosynthesis and necroptosis.

**Table 4:** Enrichment analysis and bioinformatics analysis of differentially expressed metabolites

Pathway Name	P	-log(p)	RichFactor
Glycerophospholipid metabolism	0.000	3.888	0.058
Aminoacyl-tRNA biosynthesis	0.000	3.888	0.058
Necroptosis	0.019	1.716	0.111
Sphingolipid signaling pathway	0.032	1.496	0.067
Sphingolipid metabolism	0.053	1.278	0.040
beta-Alanine metabolism	0.067	1.174	0.031
Phenylalanine, tyrosine and tryptophan biosynthesis	0.071	1.149	0.029
Histidine metabolism	0.097	1.013	0.021
Phenylalanine metabolism	0.122	0.912	0.017
Arginine and proline metabolism	0.157	0.805	0.013
ABC transporters	0.184	0.735	0.011

**Fig. 5:**
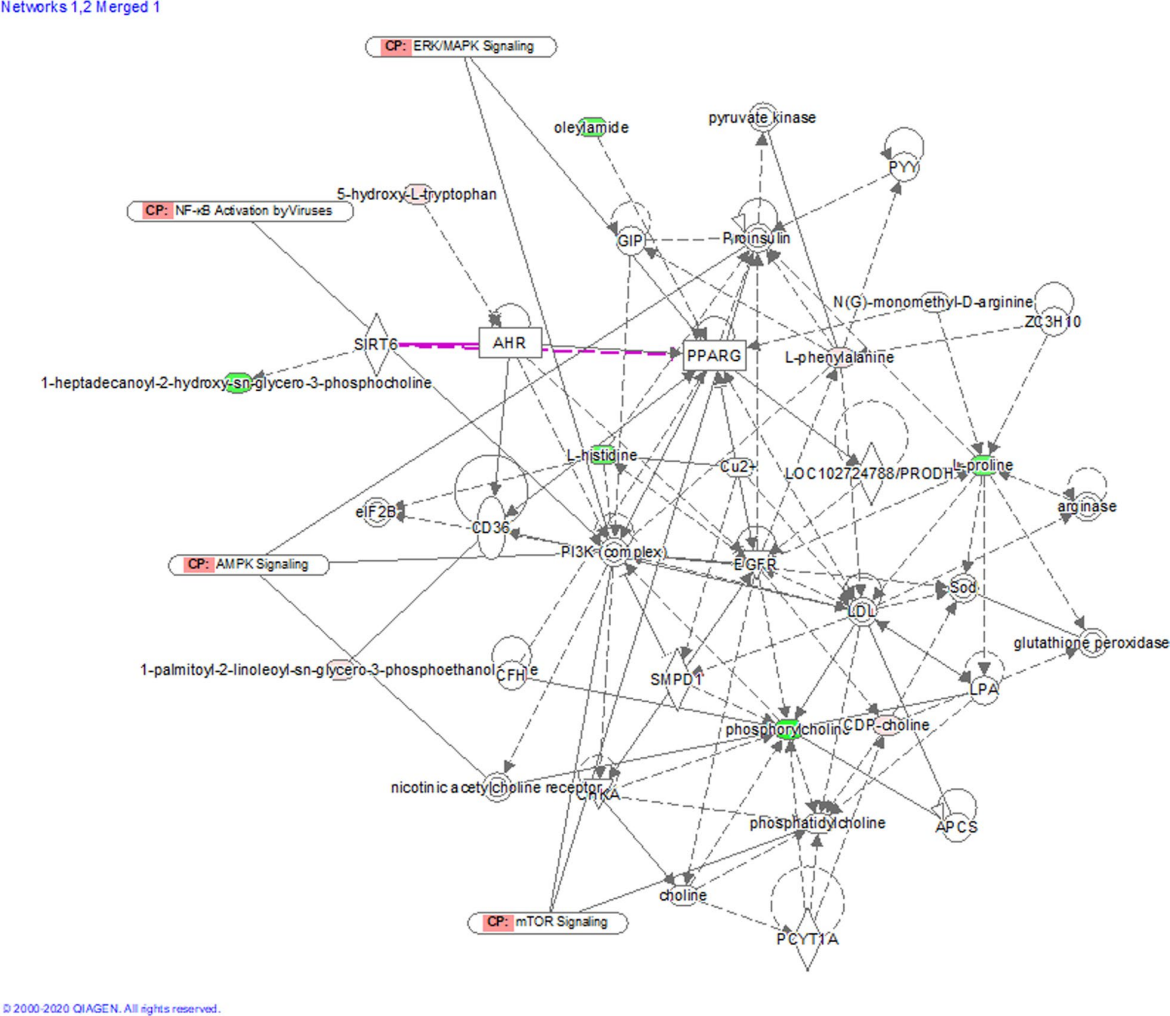
IPA network analysis of differential metabolites. Red and green nodes represent high- and low-expression levels metabolites in the sepsis group, respectively

Furthermore, the differential substance network showed that the biological pathways of significantly different metabolites included extracellular signal-regulated kinase (ERK)/mitogen-activated protein kinase (MAPK), nuclear factor-κB (NF-κB), AMP-activated protein kinase (AMPK), and mammalian target of rapamycin (mTOR). IPA suggested that lipid metabolism, cell proliferation, cell differentiation, and cell apoptosis may be potential signaling pathways through which these significantly differential metabolites may play a role (Fig. 5).

## Discussion

In the present study, non-targeted metabolomics technology was used to identify the potential biomarkers of infant sepsis at an early stage. The metabolic profiles of infants with sepsis were significantly different compared to those without. Six differential metabolites were identified, and three of them had excellent diagnostics capability (AUC > 0.7). Further, bioinformatics results showed that metabolic changes were mainly related to lipid metabolism, cell proliferation, cell differentiation and apoptosis, which provided clues for exploring mechanisms of infant sepsis in the future.

The present study found that the levels of the three metabolites (PA (8:0/14:0), PE (16:0/18:2(9Z,12Z)) and CDP-CHO) were increased in the serum of sepsis infants. PA (8:0/14:0) and PE (16:0/18:2(9Z,12Z)) are glycolipid lipids and critical components of the biofilm lipid bilayer, which are involved in metabolism and cell signaling [14]. Moreover, CDP-CHO has been reported to be an essential intermediate of phosphatidylcholine biosynthesis in membrane structures, especially phosphatidylcholine [15]. Furthermore, Jaroonwitchawan et al. used metabolomics analysis and detected elevated PE (16:0/18:2(9Z,12Z)) levels in LPS-tolerant macrophages, which is consistent with our results [16]. In addition, increased serum levels of PA (8:0/14:0) and PE (16:0/18:2(9Z,12Z)) were also identified in septic mice by lipidomic analysis [17]. But, GB Li et al. [18] study showed opposite results that a decreasing tendency of PE (16:0/18:2(9Z,12Z)) was observed in sepsis patients, similar with this study. The contradiction could be related to the heterogeneity of the subjects, which the age of cases enrolled in GB Li study ranging from 15 days after birth to 13 years old. It speculated that the differences of dietary structure and liver function between neonates and young children may affect the metabolism in physiologic and pathophysiologic conditions.

In the current study, correlation analysis showed a negative relationship between PE (16:0/18:2(9Z,12Z)) and sepsis indicators such as PCT, suggesting that PE (16:0/18:2(9Z,12Z)) may be a potential biomarker to predict severe infection. Langley et al. [19] represented the metabolites of patients died of sepsis differed markedly from those of survival patients, and emphasized the predictive value of lipid metabolites for death in patients with sepsis. In addition, the finding of this study was again reinforced by a study of lipid levels in children with sepsis, presenting a positive relationship between lipid levels and infection severity [20]. The relationship of lipids metabolism to sepsis and the exact molecular mechanism deserves further study.

SM (d18:0/16:1(9Z)) is a sphingolipid substance on the membranes of cells that contributes to the structural integrity of those cells. It is also an essential component of signal transduction modules of critical biological reactions, such as cell division, differentiation, gene expression, and apoptosis [21, 22]. This work found that SM (d18:0/16:1(9Z)) levels decreased significantly in the sepsis group, consistent with the previous study by Winkler et al. [23], concluding that SM (d18:0/16:1(9Z)) is an accurate predictor for septic mortality [24].

Phosphocholine is an essential intermediate in lecithin synthesis and participates in various enzymatic reactions. Hecker et al. [25] found that phosphocholine can effectively control the release of ATP-mediated interleukin-1 β (IL-1β) in human and mouse monocytes, and IL-1β is essential in activating the inflammatory responses in sepsis. In this study, decreased expression of phosphocholine was observed in infants with sepsis. Therefore, we hypothesized that the activation of inflammatory response in infants with sepsis might be related to the decrease of endogenous phosphocholine levels. Lysophosphatidylcholine(LysoPC), generated from phosphatidylcholine(PC), may play a role as regulator of immune function [26]. The level of LysoPC decreased in sepsis group, compared with control group (Table 2). A similar result was also found in Drobnik’s research [27], and they reported that the molar ratio of LysoPC and PC, which was a reflection of enzymatic reaction for the lipid generation, can strongly predict sepsis-related mortality. Thus, there is a possibility that LysoPC-PC ratio might be a promising index to predict the outcome of sepsis, and more critical septic cases should be included in future study. Combined with the results of lipidomic analysis of PA(8:0/14:0), PE(16:0/18:2(9Z,12Z)), CDP-CHO and SM(d18:0/16:1(9Z)), we speculated that the changes of lipid metabolites in serum of septic infants might be related to the destruction of the cell membrane during inflammatory response induced by infection.

Besides these lipid metabolites detected in present study, a few amino acids have also been found to be different between the two groups. Phenylalanine, an essential amino acid, was found to be elevated in sepsis group. Another study conducted in adult septic patients, similarly to our results [28]. Furthermore, it indicated that Phenylalanine was found to have the ability to identify high risk patients and predict the mortality related to sepsis. While the mechanism of this change is still unclear, some researchers noted that the increasing concentration of phenylalanine might be related to insufficient tissue perfusion, increased insulin resistance, dysfunctional energy production and the large consumption of tetrahydrobiopterin [29–31]. The serum level of another amino acid, histidine decreased in sepsis group in this study. Histidine is essential for infants and may be able to inhibit the expression of pro-inflammatory cytokines [32]. The present results are consistent with another research carried out in adult patients suffered sepsis [16].

There are some limitations of classical indicators in neonatal sepsis: the WBC is not a very useful diagnostic marker because the specificity is low [33]; there are many conditions that cause PCT to increase falsely, such as nonspecific elevation in healthy newborns, prematurity, intracranial hemorrhage, birth asphyxia and neonatal hypoxemia [34], that showed limited value in the diagnosis of early-onset sepsis in neonates; the specificity of CRP is affected by non-infection conditions including hemolysis, tissue injury, surgery, stressful delivery, etc. [35] Correlation analysis revealed that prolylhydroxyproline, PE(16:0/18:2(9Z,12Z)) and CDP-CHO were associated with CRP or PCT, in addition, ROC analysis demonstrated that the three metabolites together improved the diagnostic accuracy. It is still necessary to conduct more studies with larger samples to verify diagnostic and prognostic values of metabolites.

Further, pathway analysis indicated that the glycerophospholipid metabolism, aminoacyl-tRNA biosynthesis and necroptosis might play an important role during sepsis. Subsequently, metabolite network analysis suggested that changes in metabolites were related to classic stress and inflammatory pathways such as ERK/MRPK, NF-κB, AMPK, and mTOR. Therefore, in addition to identifying potential markers of infant sepsis, the present study provides clues to the development of sepsis in infants.

Most of subjects in this study were neonates, differed from the previous pediatric cohort study [18]. All the subjects were term infants, and their mothers had no metabolic factors, which would influence the metabolite detection. Therefore, the results were more reliable. This study suggested clinical value of metabolomics in neonatal sepsis. In the future, the application and mechanisms of metabolites in neonatal infection need to be investigated.

Metabolomics is a valuable tool for diagnosis of sepsis in adults [36]. Compared with adults, infants with the dominantly milk-based diet and with little co-morbidities, the results of metabolites in those are more stable and reliable. The present report describes a clear clustering of the metabolomes in infants with sepsis, highlight the potential metabolites as early biomarkers for infant sepsis. A further strength of our study lies in study population, which studies selected infants or neonates are still scant.

A limitation of this study concerns the relatively limited number of cases. Compare with the number of cases in previously published study [37] on metabolomics in diagnosis of preterm early onset sepsis, the number of included cases in the present study was similar. Another weakness of this report is lack of evidence for association between metabolites and adverse outcomes. Finally, the results need to be validated and improved by further studies.

## Conclusion

In infant sepsis, six differential metabolites identified by LC-MS analysis, PA (8:0/14:0), PE (16:0/18:2(9Z,12Z)), CDP-CHO, SM (d18:0/16:1(9Z)), P-CHO and prolylhydroxyproline, showed significant differences between sepsis and control groups. Combined of PE (16:0/18:2(9Z,12Z)), CDP-CHO and prolylhydroxyproline showed good AUC values. Furthermore, these three metabolites were associated with CRP or PCT levels. The present results indicated that these metabolites have potential as biomarkers for early diagnosis of infantile sepsis.

## Supplementary Information

**Additional file 1: Table S1. **26 differential metabolites identified by VIP>1 and P<0.05.

**Additional file 2: Table S2.** Statistics of markers.

**Additional file 3: Table S3.** Confusion matrices of 6 lipid metabolites.

**Additional file 4: Figure S1.** Volcano plots and heatmap of differential metabolites.

**Additional file 5: Appendix S1.** MS2 fragment ion spectrum of differential metabolites.

## Data Availability

Data are available upon contact the author Li Wang at wlsjtu@163.com.

## Abbreviations

ROC: Receiver operating characteristic
AUC: Area Under the Curve
PROM: Premature rupture of membrane
GDM: Gestational diabetes mellitus
WBC: White blood count
CRP: C-reactive protein
PCT: Procalcitonin
ERK: Extracellular signal-regulated kinase
MAPK: Mitogen-activated protein kinase
NF-κB: Nuclear factor-κB
AMPK: AMP-activated protein kinase
mTOR: Mammalian target of rapamycin
LC-MS: Liquid chromatography-mass spectrometry
QC: Quality control
PLS-DA: Partial least squares discriminant analysis
KEGG: Kyoto Encyclopedia of Genes and Genomes
IPA: Ingenuity pathway analysis
PA: Phosphatidic acid
PE: Phosphatidyl ethanolamine
CDP-CHO: Cytidine 5'-diphosphocholine
SM: Sphingomyelin
P-CHO: Phosphorylcholine
