# Supplementary figures and images for: Discrimination of serum metabolomics profiles in infants with sepsis, based on liquid chromatography-mass spectrometer
Source: BMC Infect Dis. 2023 Jan 23;23:46. doi: 10.1186/s12879-023-07983-w (PMC9872383; doi:10.1186/s12879-023-07983-w)

**Figure S1.** Volcano plots and heatmap of differential metabolites.


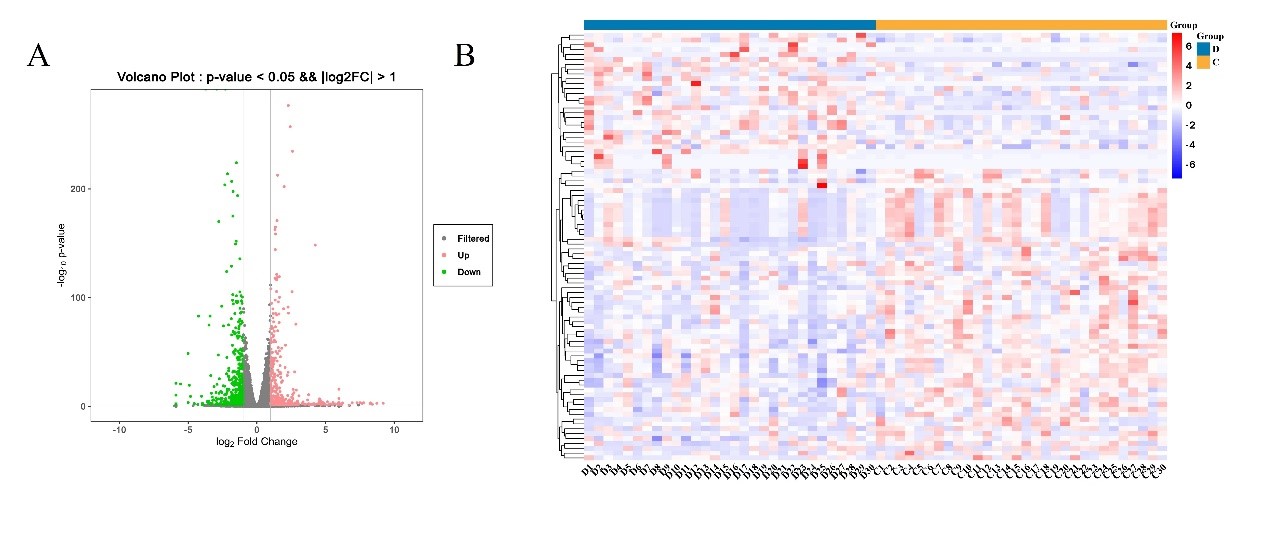

Supplement: Supplementary file 4 — Additional file 4: Figure S1. Volcano plots and heatmap of differential metabolites. [file 12879_2023_7983_MOESM4_ESM.docx]
