# Supplementary material for: Discrimination of serum metabolomics profiles in infants with sepsis, based on liquid chromatography-mass spectrometer
Source: BMC Infect Dis. 2023 Jan 23;23:46. doi: 10.1186/s12879-023-07983-w (PMC9872383; doi:10.1186/s12879-023-07983-w)

Prolylhydroxyproline


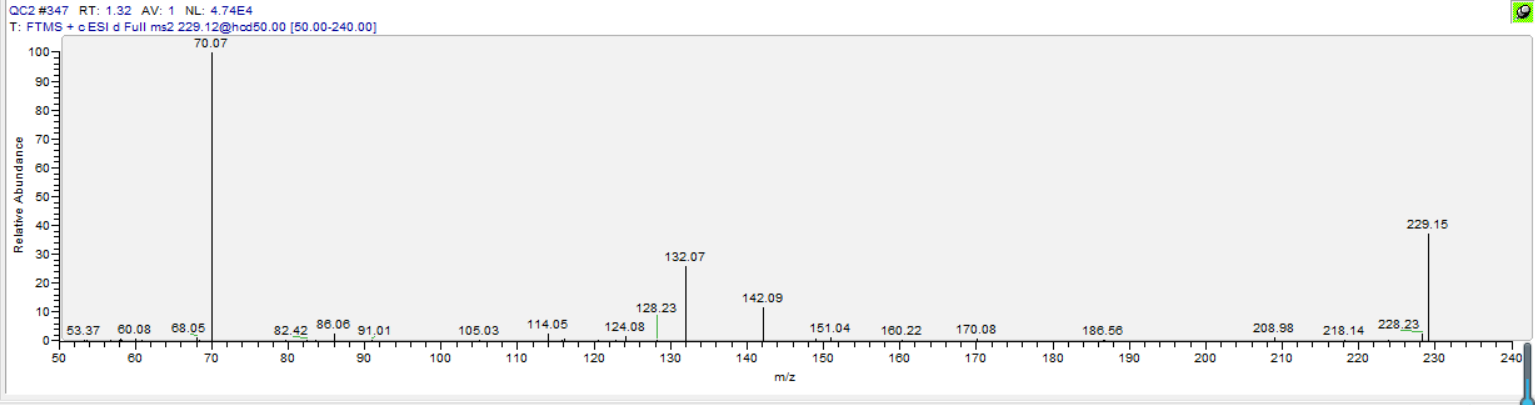


Phosphocholine


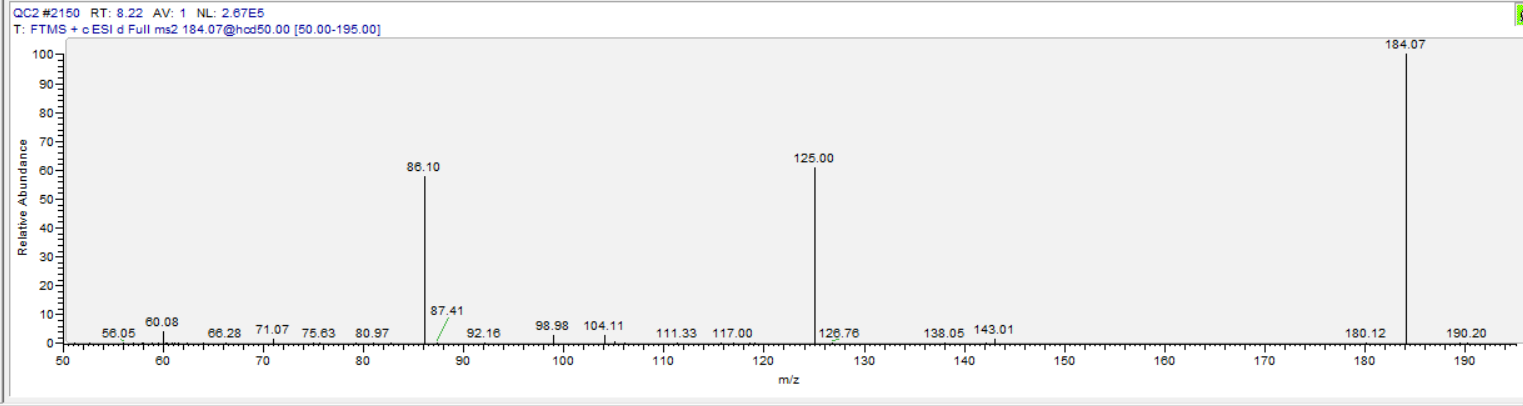


PE(16:0/18:2(9Z,12Z))


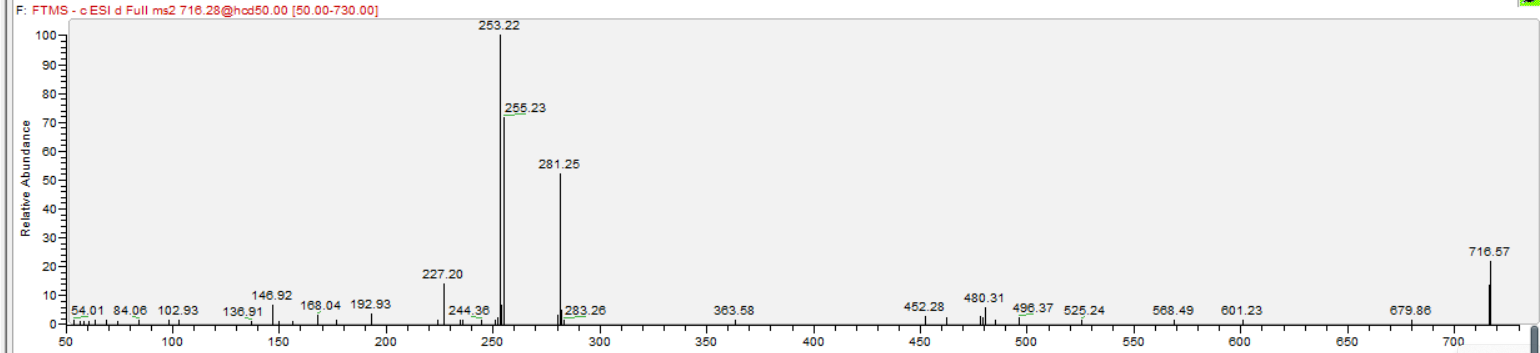


Cytidine 5'-diphosphocholine


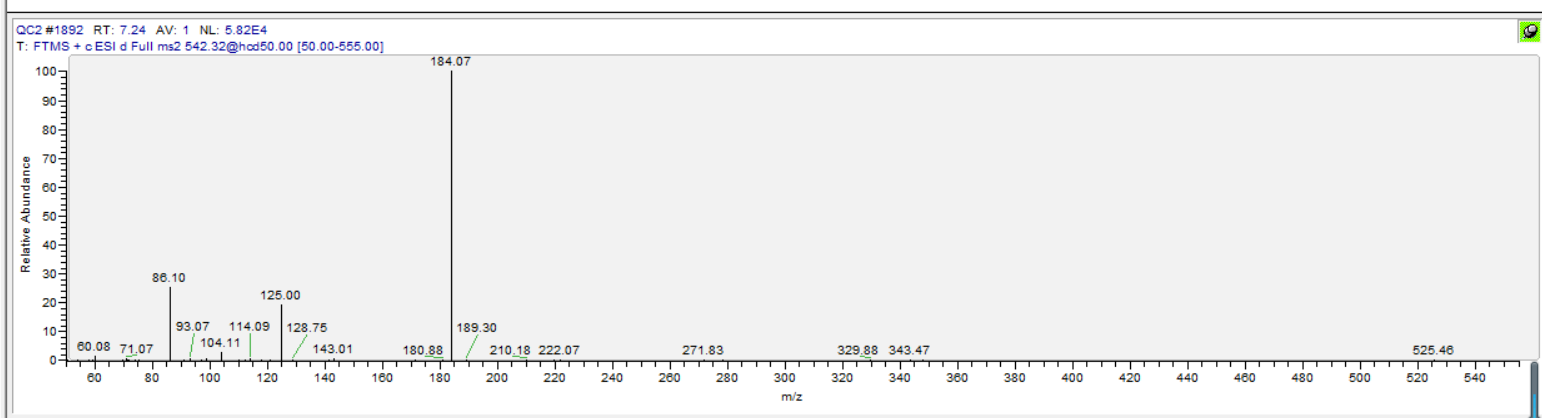


SM(d18:0/16:1(9Z))


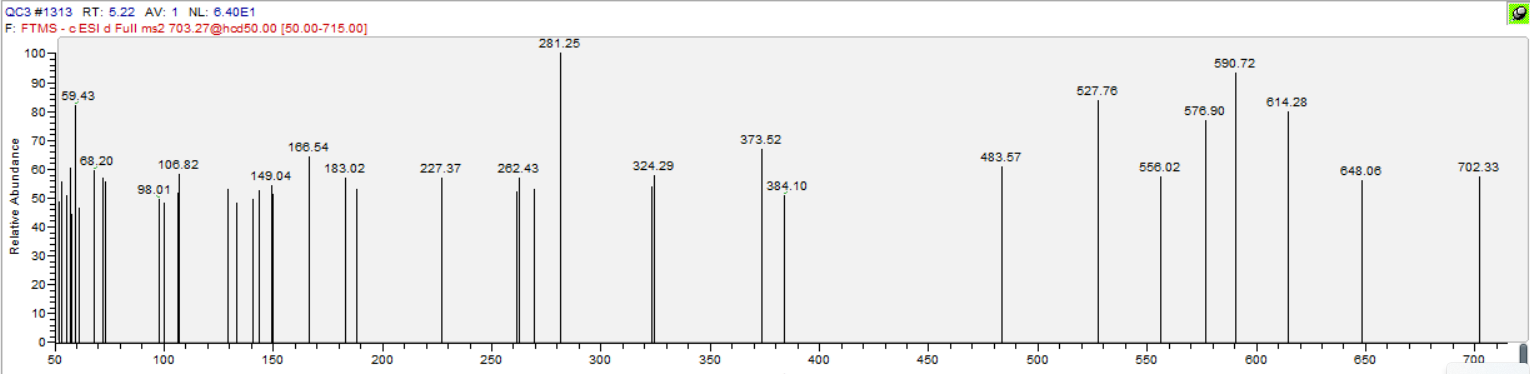


PA(8:0/14:0)


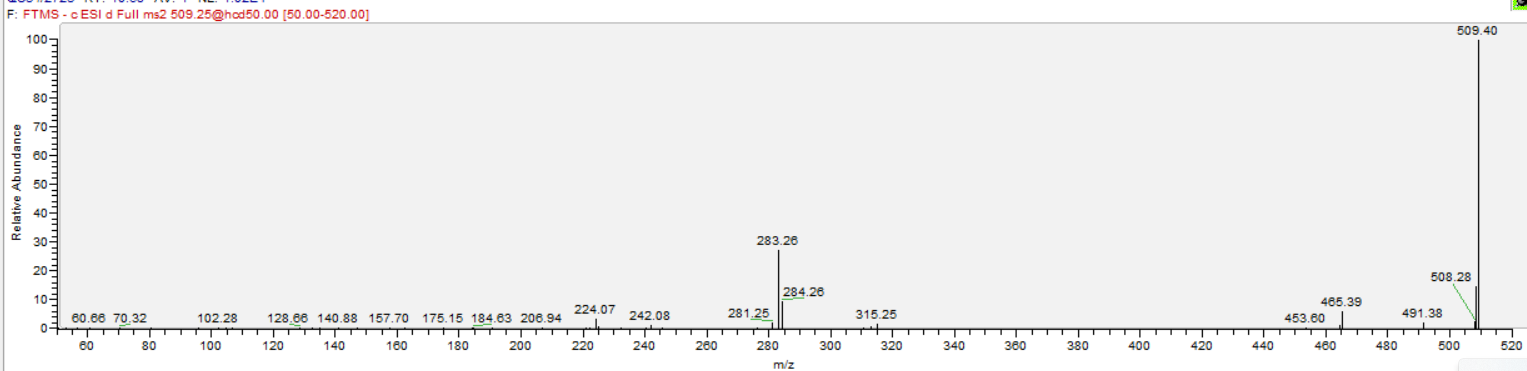


3-Dehydro-6-deoxoteasterone


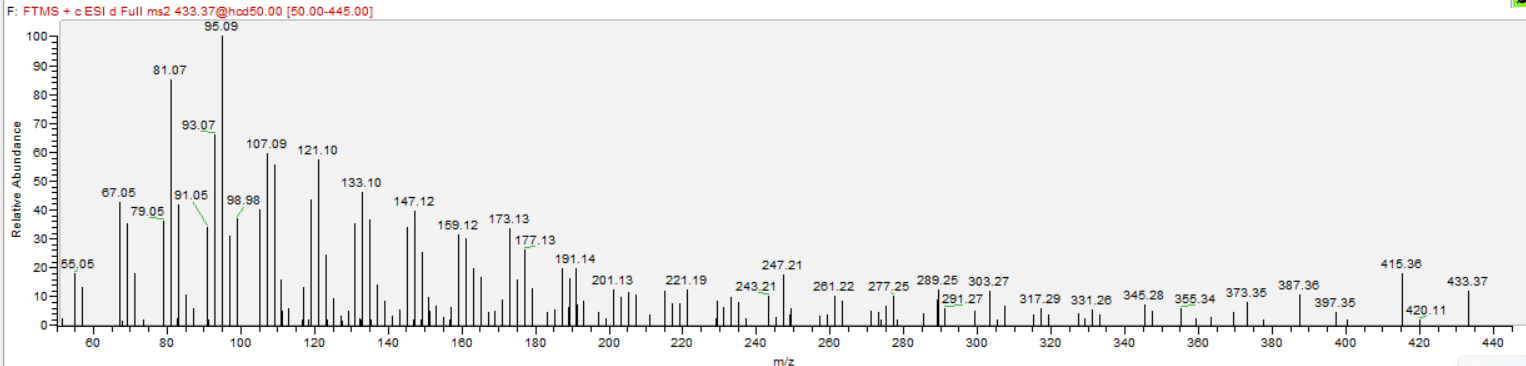


Capsiamide


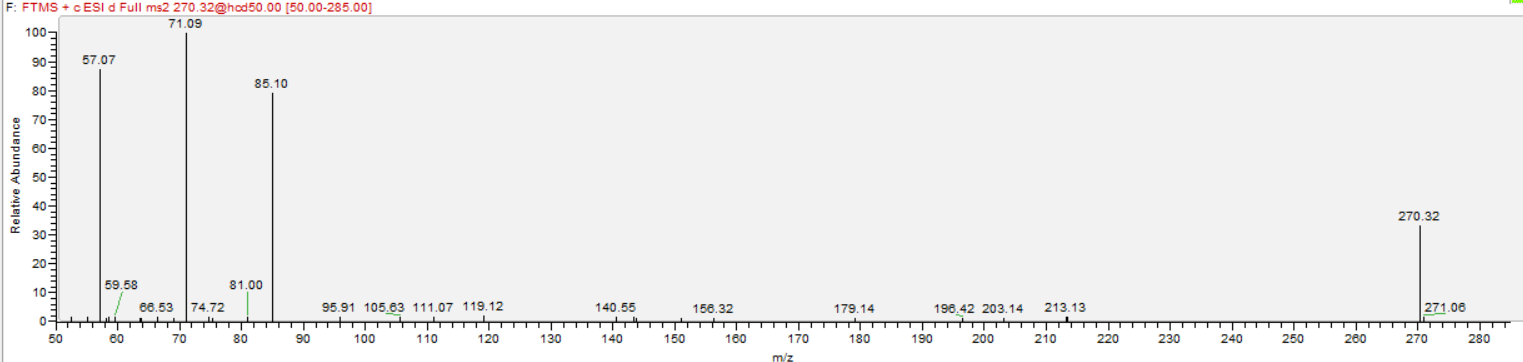


Ethyl oleate


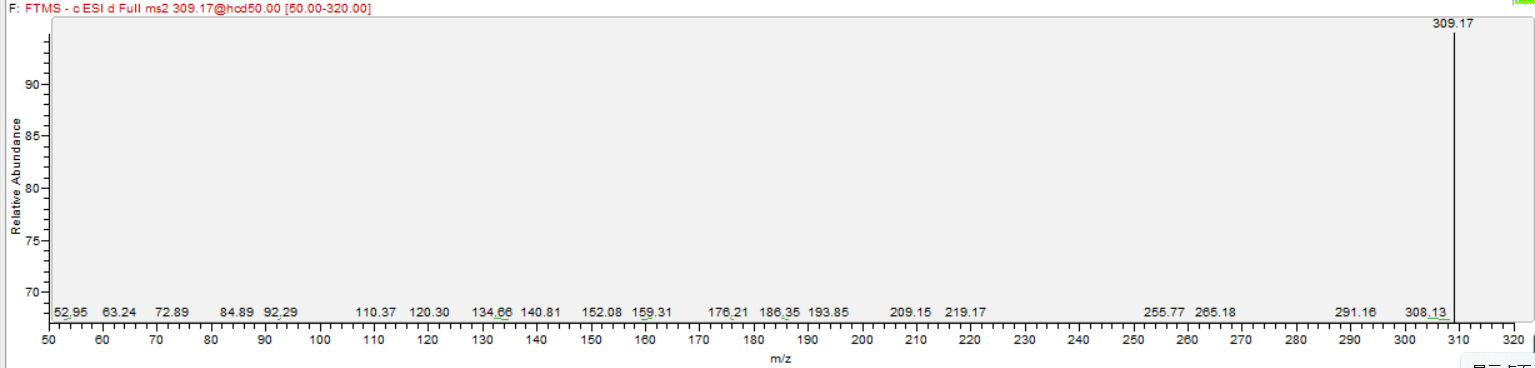


Tazobactam


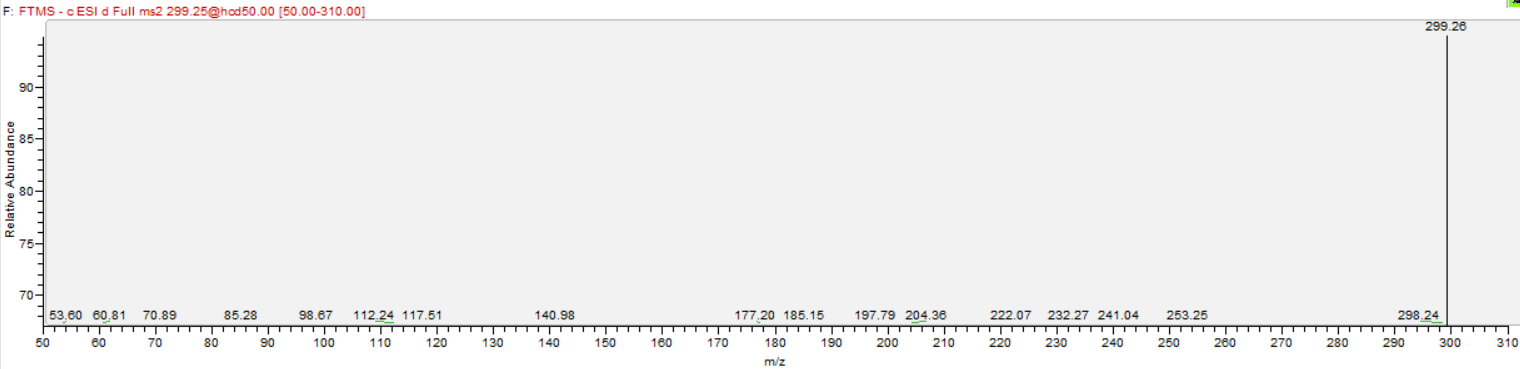


Cefepime


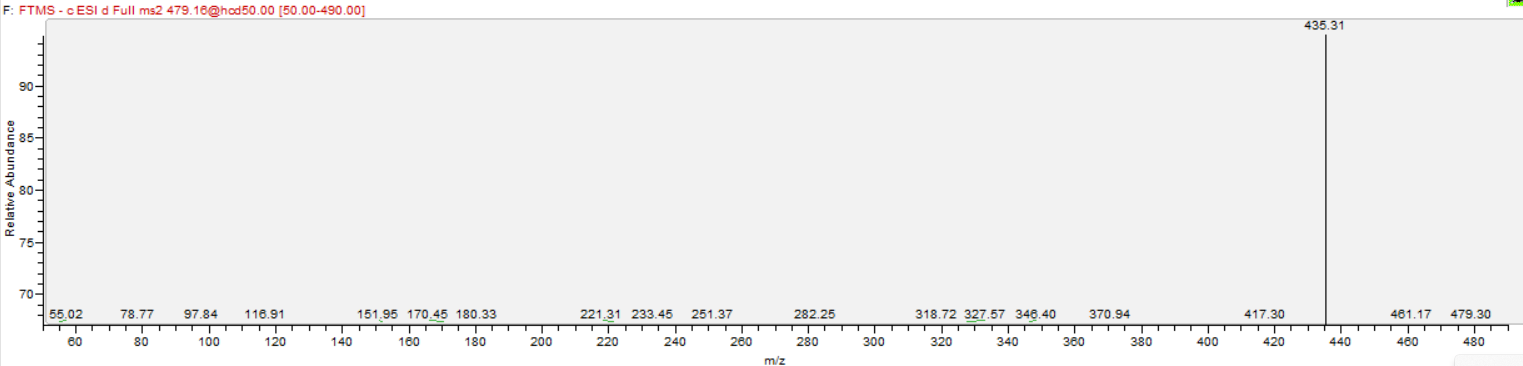


LysoPC(17:0)


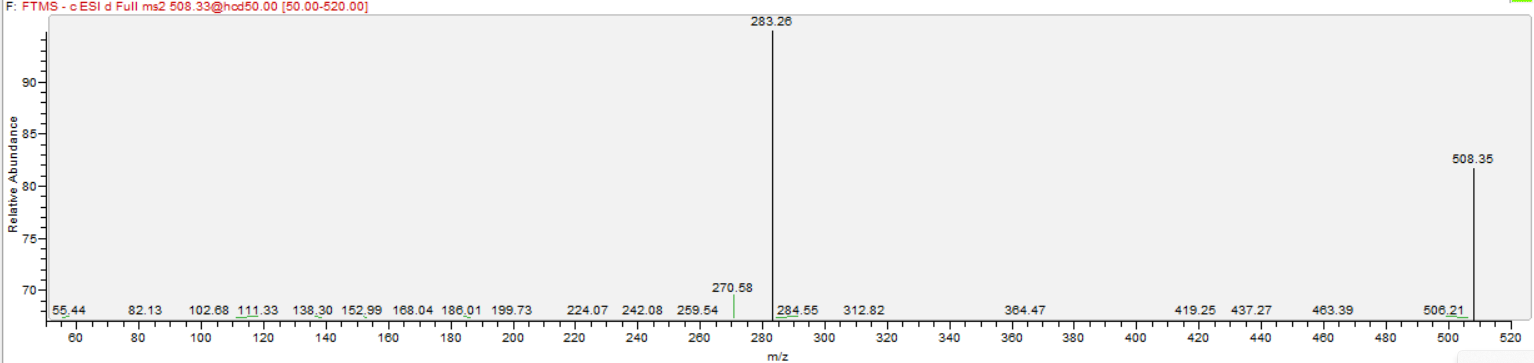


2-lauroleic acid


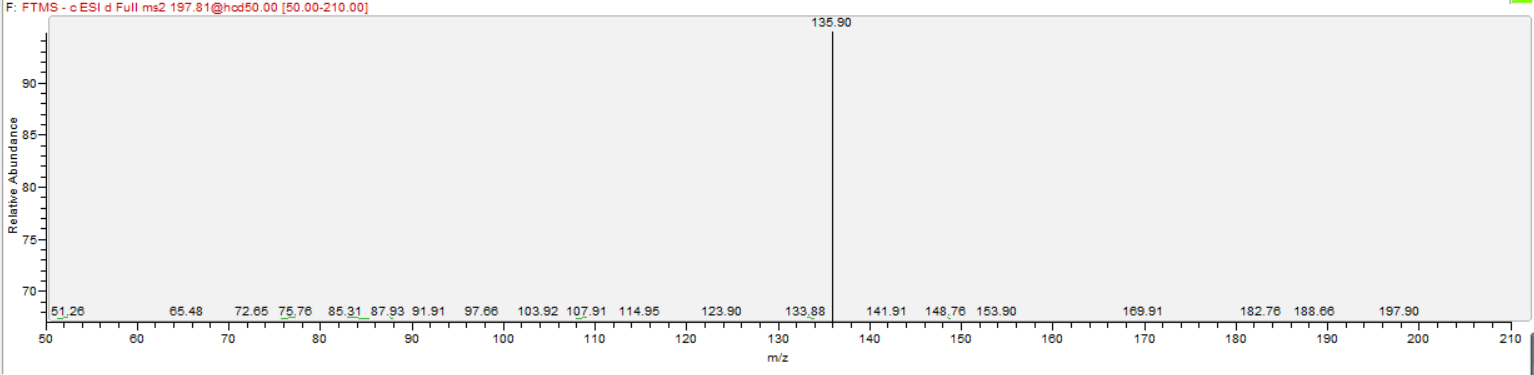


PE(16:0/18:2(9Z,12Z))


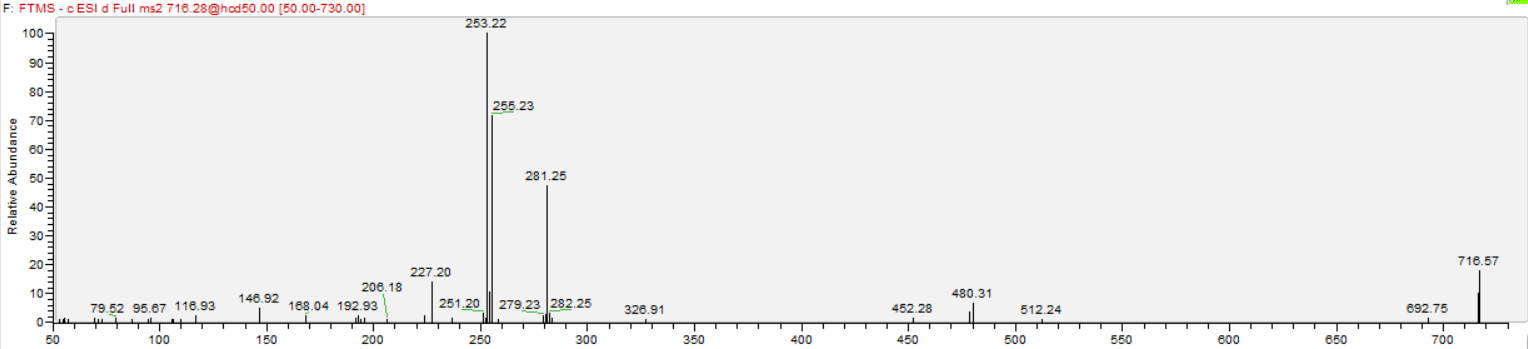


Capsiamide


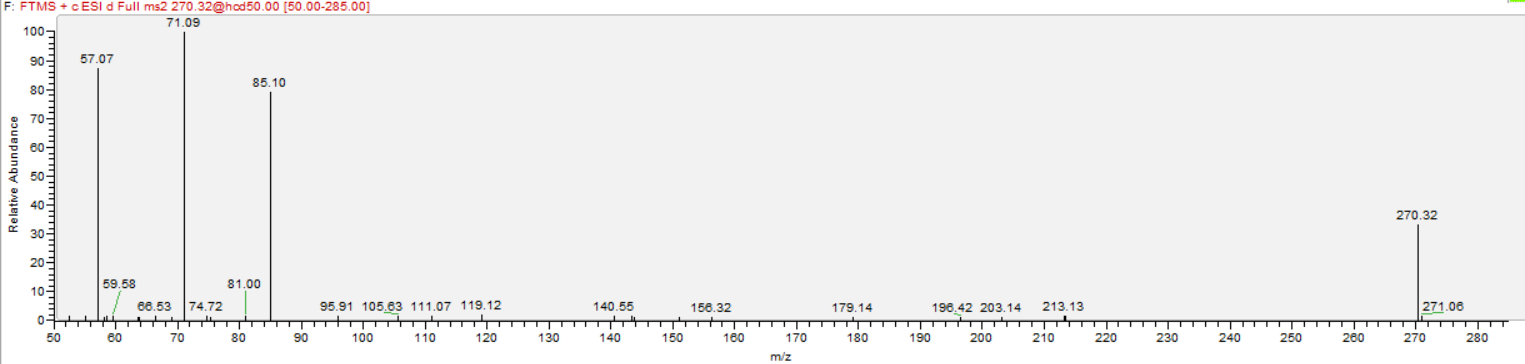


Piperidine


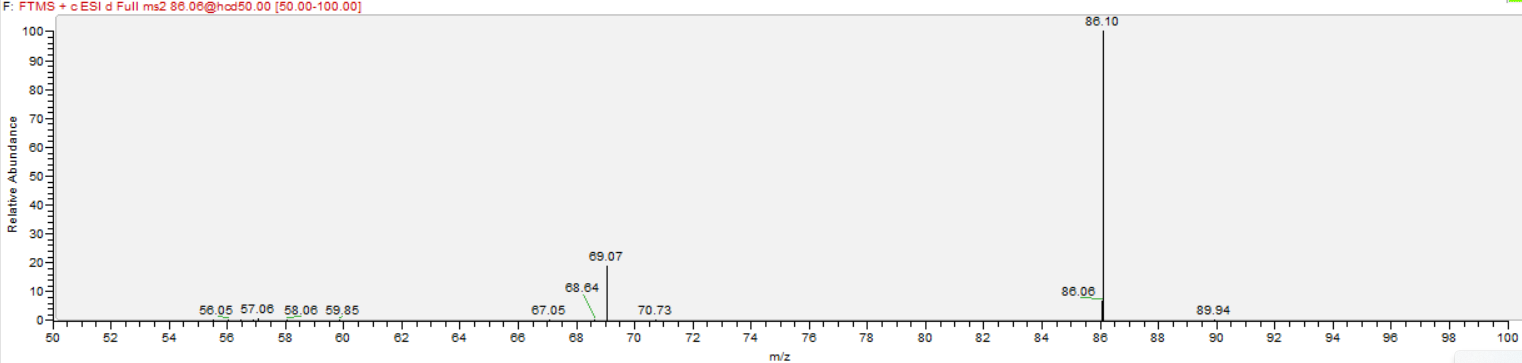


PE(16:0/0:0)


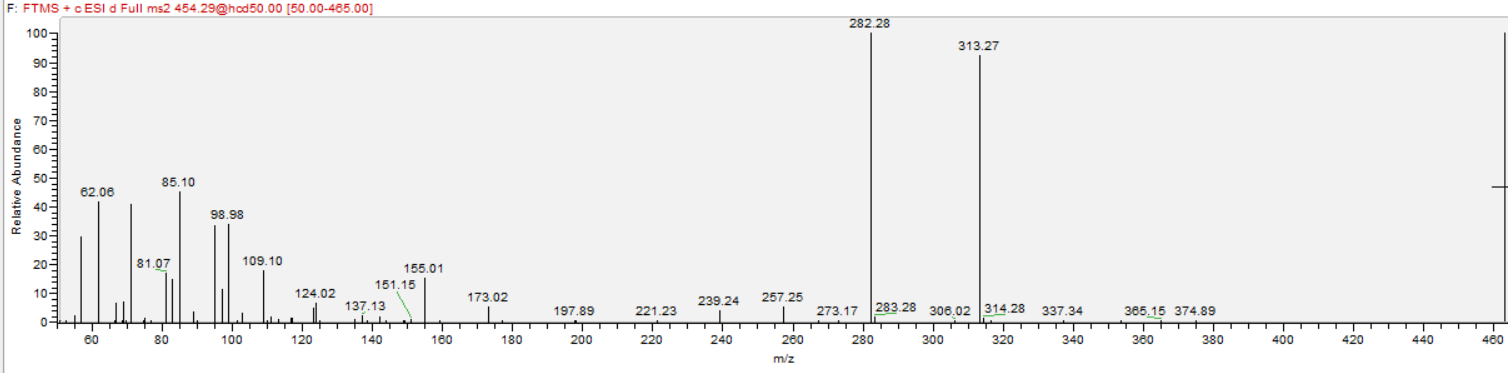


LysoPC(20:3(5Z,8Z,11Z)/0:0)


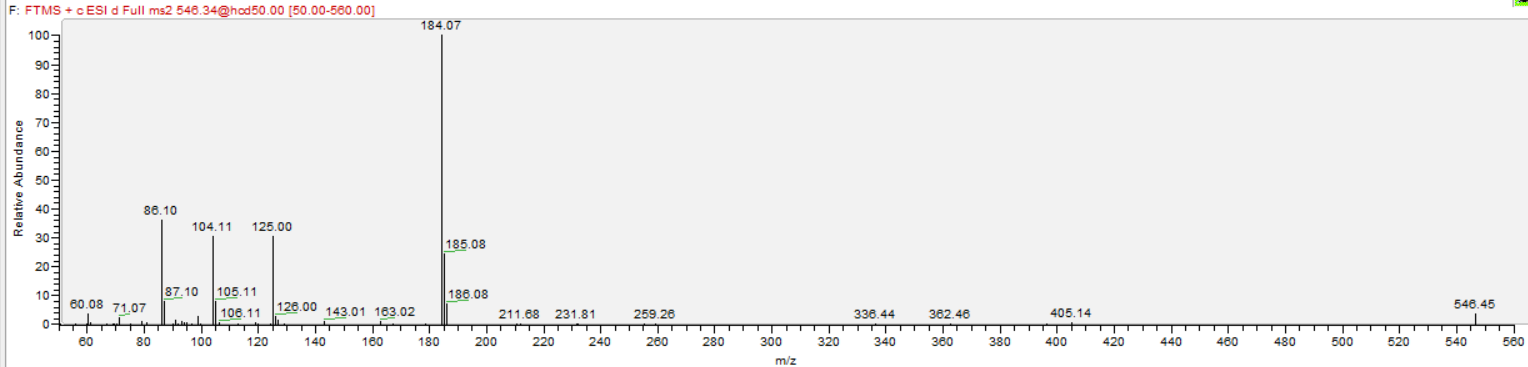


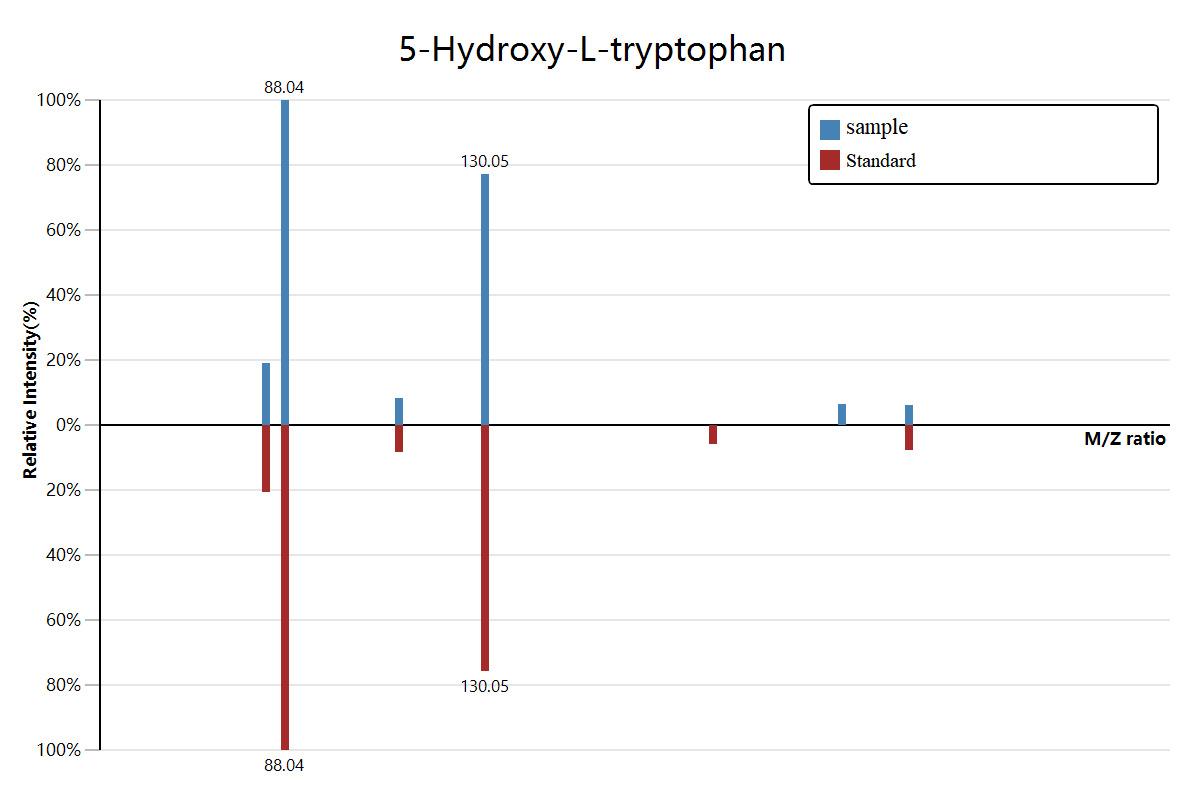

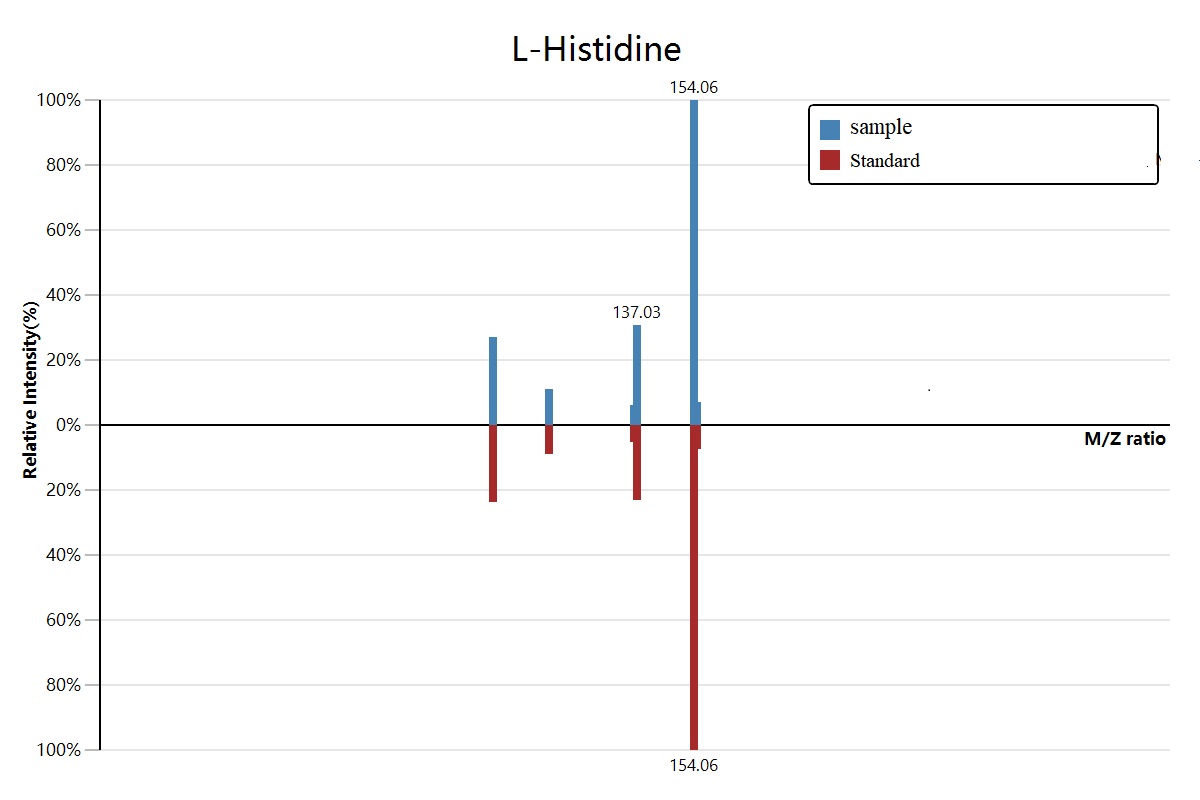

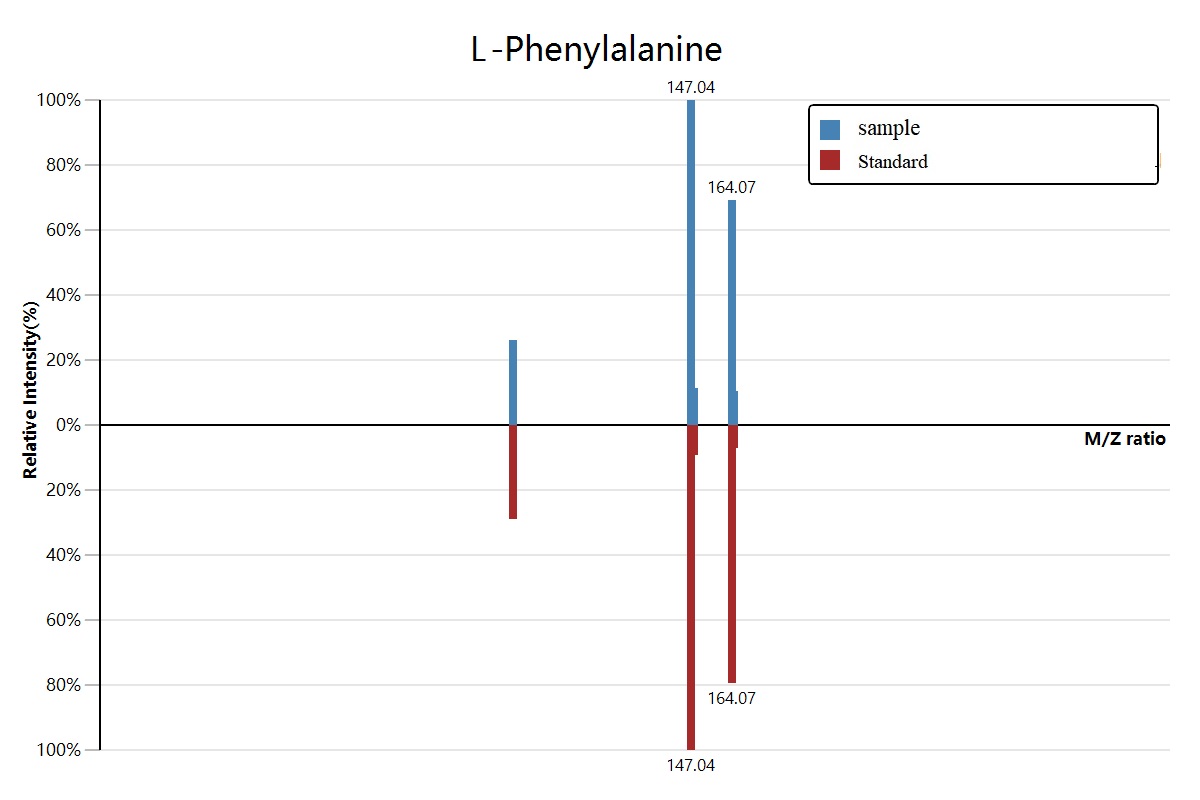

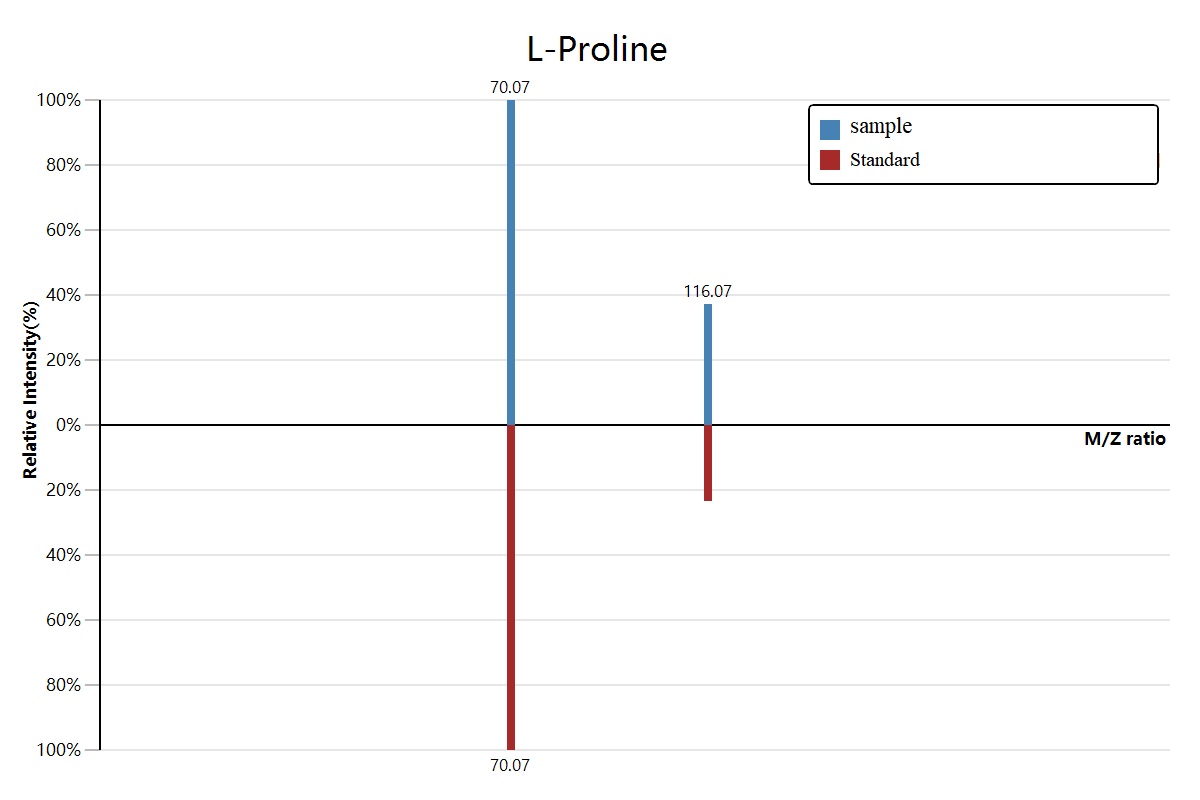

Supplement: Supplementary file 5 — Additional file 5: Appendix S1. MS2 fragment ion spectrum of differential metabolites. [file 12879_2023_7983_MOESM5_ESM.docx]
